# Barriers and Facilitators for Type-2 Diabetes Management in South Asians: A Systematic Review

**DOI:** 10.1371/journal.pone.0136202

**Published:** 2015-09-18

**Authors:** Tanveer Sohal, Parmjit Sohal, Kathryn M. King-Shier, Nadia A. Khan

**Affiliations:** 1 Department of Medicine, University of British Columbia, Vancouver, British Columbia, Canada; 2 Department of Family Medicine, University of British Columbia, Vancouver, British Columbia, Canada; 3 Faculty of Nursing, University of Calgary, Calgary, Alberta, Canada; 4 Department of Community Health Sciences, University of Calgary, Calgary, Alberta, Canada; 5 Department of Medicine, University of British Columbia, Vancouver, British Columbia, Canada; 6 Center for Health Evaluation for Outcomes Sciences, St. Paul’s Hospital, Vancouver, British Columbia, Canada; University of Tolima, COLOMBIA

## Abstract

**Objective:**

Although South Asian populations have among the highest burden of type 2 diabetes in the world, their diabetes management remains poor. We systematically reviewed studies on South Asian patient’s perspectives on the barriers and facilitators to diabetes management.

**Methods:**

We conducted a literature search using OVID, CINHAL and EMBASE (January, 1990 –February, 2014) evaluating the core components of diabetes management: interactions with health care providers, diet, exercise, and medication adherence. South Asian patients were self-reported as Indian, Pakistani, Malaysian-Indian or Bangladeshi origin. From 208 abstracts reviewed, 20 studies were included (19 qualitative including mixed methods studies, 1 questionnaire). Barriers and facilitators were extracted and combined using qualitative synthesis.

**Results:**

All studies included barriers and few facilitators were identified. Language and communication discordance with the healthcare provider was a significant barrier to receiving and understanding diabetes education. There was inconsistent willingness to partake in self-management with preference for following their physician’s guidance. Barriers to adopting a diabetic diet were lack of specific details on South Asian tailored diabetic diet; social responsibilities to continue with a traditional diet, and misconceptions on the components of the diabetic diet. For exercise, South Asian patients were concerned with lack of gender specific exercise facilities and fear of injury or worsening health with exercise. Patients reported a lack of understanding about diabetes medication management, preference for folk and phytotherapy, and concerns about the long-term safety of diabetes medications. Facilitators included trust in care providers, use of culturally appropriate exercise and dietary advice and increasing family involvement. Overall themes for the barriers included lack of knowledge and misperceptions as well as lack of cultural adaptation to diabetes management.

**Conclusion:**

Diabetes programs that focus on improving communication, addressing prevailing misconceptions, and culture specific strategies may be useful for improving diabetes management for South Asians.

## Introduction

South Asians represent approximately one-fifth of the global population and have a disproportionately higher incidence of type-2 diabetes mellitus. Diabetes occurs at 50% higher rates in South Asian patients compared to the general population [1]. Diabetes develops 5 to 10 years earlier and is one of the principle causes of premature heart attack and death in this population [1, 2]. Multiple randomized controlled trials identified pharmacological and non-pharmacological strategies to substantially reduce the risk of diabetes related complications and premature mortality. These effective strategies for chronic diabetes management are incorporated into diabetes guidelines and include: ongoing diabetes monitoring by a health care provider, adherence to a diabetic diet, exercise and medical therapy [3].

Despite clear evidence that diabetes management (dietary changes, regular exercise, and adherence to appropriate medications) leads to a 53–63% reduction in complications and a 46% reduction in mortality [4,5], diabetes management and control remain poor in South Asian patients. South Asians are less likely to exercise or follow a healthy diet compared to the general population [6]. Among South Asian patients with diabetes prescribed oral hypoglycemic agents, ACE inhibitor and statin therapy, only 40–45% were adherent to these medications [7]. North American studies identified that the majority (55%) of South Asian patients were above recommended A1C targets (target A1C ≤7%, the standard measure for the average level of blood sugar over the past 2–3 months), 36% were above blood pressure targets and 58% were above lipid level targets for diabetes [8]. South Asian patients were 24% less likely to achieve these critical targets than White patients [9] and had 60–113% higher A1C levels compared with the general population [10–12]. According to the International Diabetes Federation (IDF), the global health expenditure of diabetes including diabetes management and costs attributed to complications was $376 billion (USD) in 2010. This cost is expected to exceed $490 billion (USD) by 2030. Understanding patient level barriers and facilitators perceived or experienced underlying poor diabetes management is essential to inform the development of effective diabetes education and management programs to reduce risk of diabetes-related complications.

To help clarify the factors influencing diabetes management, we conducted a systematic review of qualitative and mixed methods studies investigating patient’s views, attitudes or beliefs on barriers and facilitators in the core components of diabetes management: interactions with health care workers, diet, exercise and medication adherence in South Asian patients with type 2 diabetes.

## Methods

### Data Sources and Searches

We conducted a literature search investigating patient reported barriers and facilitators in South Asian patients with type 2 diabetes. With a medical librarian, we conducted a search using OVID, CINHAL and EMBASE from January 1, 1990 to February 1, 2014. We also hand searched review articles on barriers and facilitators. We used the following medical subject headings and free text terms: India AND Diabetes Mellitus, Type-2 AND Management.mp, India AND Diabetes Mellitus, Type 2 AND Manage$.mp, India AND Diabetes Mellitus, Type 2 AND Intervention.mp, Pakistan AND Diabetes Mellitus, Type 2 AND Management.mp, Pakistan AND Diabetes Mellitus, Type 2 AND Manage$.mp, Pakistan AND Diabetes Mellitus, Type 2 AND Intervention.mp, Bangladesh AND Diabetes Mellitus, Type 2 AND Management.mp, Bangladesh AND Diabetes Mellitus, Type 2 AND Manage$.mp, and Bangladesh AND Diabetes Mellitus, Type 2 AND Intervention.mp.

### Study Selection

We included qualitative studies that used either interviews or focus groups to examine barriers and facilitators to type-2 diabetes management as well as mixed methods quantitative studies that included surveys and interviews. South Asian patients were defined as of self-reported Indian, Pakistani, Malaysian-Indian or Bangladeshi origin. Studies with pediatric populations, type-1 diabetes, gestational diabetes, were excluded. Studies involving views from only health care workers and studies published before 1990 were also excluded. There was no language restriction. There were 500 records identified through the database searches, and 377 records after the duplicates were removed. Finally, there were 208 abstracts and full text articles assessed for eligibility. From 208 abstracts or full text articles reviewed, 20 studies were included in this review (19 qualitative or mixed methods with a qualitative component). Excluded studies consisted of 67 patient management studies, 45 chronic disease prevention studies, 36 studies using health and safety units, 32 studies of health care workers, 10 studies evaluating culturally tailored interventions, 12 based on patient education only, 11 pediatric, and 3 surgical studies.

### Study Quality Assessment and Data Extraction

We assessed the quality and risk of bias of qualitative studies using CASP (Critical Appraisal Skills Program) [13], NICE-SCIE (National Institute for Health and Clinical Excellence-Social Care Institute of Excellence) criteria [14] as well as the NIH quality assessment tool for observational and cross-sectional studies [15]. The quality appraisal criteria for qualitative studies included: study purpose, design, type of reasoning involved, thinking of theoretical perspective of the researcher, saturation sampling, constant comparison, inductive/collaborated findings, decision trail developed and rules of analysis reported, process of transforming data into themes/codes, and reported triangulation (Table 1).

**Table 1:** Summary of Studies by Country.

Country (Number of Studies)	Ethnicity	Sample Size	Female (%)	Age	Duration of Diabetes	Average Age
India (5)	Indian Malaysian	1215	38.3	20–80	6.5 >10 5.95+4.42	46.5
England (8)	BangladeshPakistani White Kashmiri Afro-Caribbean	533	39.9	21–81	2–15	52.9
Scotland (3)	BangladeshiIndian Pakistani	123	65	30–71	0–16	47
Norway (2)	Pakistani	41	73	38–7	0.5–15	52
USA (2)	BangladeshiIndian	77	48.5	54–60	>18	57

Data were collected by two reviewers in duplicate (TS, NK) on patient’s views, beliefs, understanding, attitudes and experiences on barriers and facilitators within diabetes management categories: healthcare worker interactions, exercise, diet, and medication adherence. We did not include other components of diabetes care including ophthalmology visits, foot care, and monitoring as there were insufficient data for these topics in the primary studies.

### Data Synthesis and Analysis

We constructed evidence tables of the barriers and facilitators organized by diabetes management component. Study findings were also stratified among those studies where the participants lived in Western countries, and those living in South Asian countries.

For the data synthesis, we drew from Schutz’s first, second and third order qualitative synthesis framework for meta-ethnography [16]. A meta-ethnographic analysis is interpretive rather than purely aggregative. We first reviewed the direct quotations of participant’s views or beliefs presented in each study (first-order constructs) and the primary study author’s interpretations of the data (second-order constructs). Two authors (TS, NK) coded the first and second order constructs independently. There was a substantial amount of consistency between the coders and any conflicts were resolved by using an iterative process and discussion to reach consensus. We then determined the themes across the first and second order constructs to develop third order constructs. Two reviewers (TS, NK) met to analyze and discuss the first-and second-order constructs, resulting in continuous development and refinement of third-order constructs. These constructs were interpreted and organized into overall diabetes management barriers [17].

## Results

### Primary Study Characteristics

**Fig 1:**
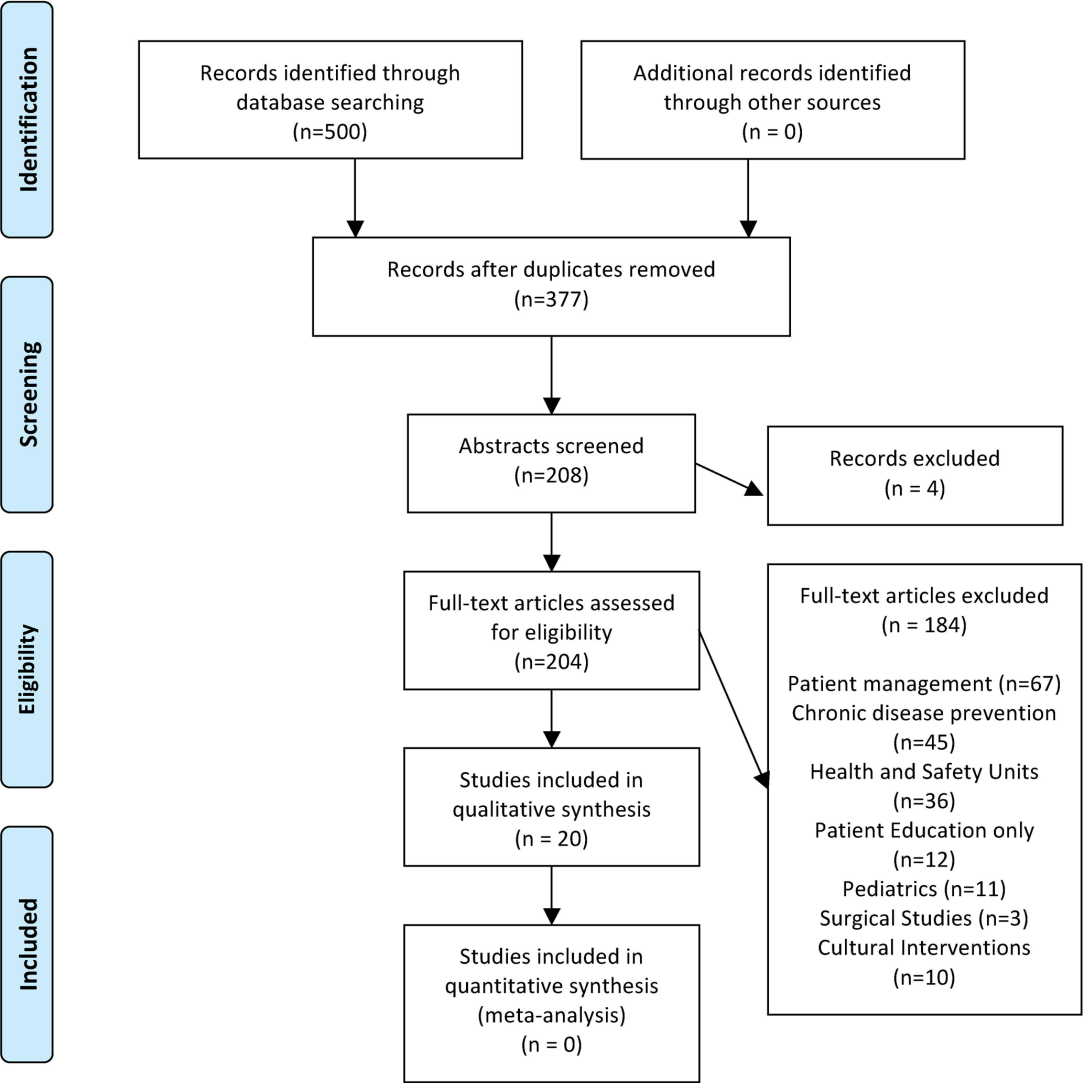
Participant Flow Diagram. *From:* Moher D, Liberati A, Tetzlaff J, Altman DG, The PRISMA Group (2009). *P*referred *R*eporting *I*tems for *S*ystematic Reviews and *M*eta*A*nalyses: The PRISMA Statement. PLoS Med 6(6): e1000097. doi:10.1371/journal.pmed1000097

There were a total of 20 studies (n = 1980 participants) collected based upon the search terms from the online databases (Fig 1). Studies included participants originating from Bangladesh (n = 6), Pakistan (n = 7), and India (n = 9) (Table 2). As per to Table 1, studies were conducted in England (n = 8), Scotland (n = 3), Norway (n = 2), US (n = 2), and India (n = 5). The majority of the studies were qualitative studies [18–35], one cross sectional survey [36] and one mixed methods study [37]. Most qualitative studies used in-depth interviews and considered the ethnography (What is the culture of a group of people?) or phenomenology (What is it like to have a certain experience?) of the ethnic minority populations. The majority of participants were women and ages ranged between 20–80 years. Few studies included information on the participant’s religion.

**Table 2:** Study Characteristics.

Author, year	Country	Setting	Participant Ethnicity	Typology	Sample Size	Women (%)	Age Range, years	Diabetes Duration, years	Average Age, years
Rhode 2003 [18]	England	1 Hospital, 2 Diabetes Clinics, GP or Practice Nurse	Bangladeshi	Semi-structured, focused interviews	55	67	21–81	2–15	51
Rhode 2003 [19]	England	Central Diabetes Register	Bangladeshi	In-depth interviews	12	67	43–75	Mean 6 Bangladeshi; 9 Others	59
Chacko 2003 [20]	India	3 Hospitals with diabetes treatment facilities	Malaysian	Patient observations, in-depth interviews	50	52	42–75	N/A	58.5
Meeto 2005 [21]	England	Diabetes Clinic	Pakistani, Caucasian	Semi-structured interviews	49	68 Asian 58W	20–80	>1	50
Naeem 2003 [22]	England	Diabetes Clinic	Kashmiri	Interview	106	0	>40–60+	N/A	50
Kellehar1994 [23]	England	Health Centre	Bangladeshi	Interview	40	37.5	Vary	N/A	
Stone 2005 [24]	England	GP Clinics	Indian, Caucasian	Semi-structured interviews	20 (15 SA, 5 W)	55 (60 SA, 40 W)	33–80	8.7 (SA), 11.4 (W)	56.5
Fagerli 2007 [25]	Norway	GP, Local Hospitals, group education program on diabetes	Pakistani	Semi-structured Interviews	26 (15 diabetes)	73	38–66	0–1+	52
Lawton2006 [26]	Scotland	GP, Local Community	Pakistani, Indian	Interview	23 Pakistani, 9 Indian	52 Pakistani 56 Indian	30–71	0–16+	50.5
Fagerli 2005 [27]	Norway	GP, Local Hospital	Pakistani	Semi-structured interviews	15	73	38–66	0.5–15	52
Lawton 2005 [28]	Scotland	Primary care, Community sources	Pakistani, Indian	In-depth Interviews	32 (9 Indian, 23 Pakistani)	53	30–71	0–16+	50.5
Greenhalgh 1998 [29]	England	Primary Care	Bangladeshi, 10, 9 Caucasian, 2 Afro-Caribbean	Narratives, semi-structured interviews, focus groups, and pile sorting exercises	50	34	21–80	N/A	50.5
Venkatesh 2013 [30]	US	Asian Indian organizations, stores, restaurants, relevant organizations	Indian	In-depth audiotaped interviews	30	47	54–60	>18	57
Venkataraman 2011 [31]	India	Hospital	Indian	Focus groups, semi-structured interviews	507	55.4	Mean: 54	Mean: 6.5	54
Jepson 2012 [32]	Scotland	Natural Groups (exercise, mother & baby, prayer)	Bangladeshi, Indian, Pakistani	Focus groups, In-depth interviews	59	56%	Middle-age	N/A	40
Islam 2012 [33]	US	Community Events	Bangladeshi	Focus groups	47	50	N/A	N/A	
Wilson 2010 [34]	India	GP & cardiologist offices	Indian	Formal & informal interviewsPatient observations	200	N/A	N/A	N/A	
Mendenhall 2012 [35]	India	Enrolled in cardio-metabolic Risk Reduction in South Asia Study	Indian	Interview	59	49%	Over 20	>10 (41%)	20
Patel 2012 [36]	India	Diabetes Centre	Indian	Questionnaire	399	35%	Mean: 53	5.95+4.42	53
Hawthorne 1999 [37]	England	Diabetes Center and GP run diabetic mini-clinics	Pakistani	Semi-structured interview & Questionnaire	201	53%	Mean: 53	Mean Women 5; men 7.8	53

GP refers to General Practitioner. W refers to White. SA refers to South Asian.

### Quality of Primary Studies

For the qualitative and mixed methods studies, few studies reported information on whether sampling was done to saturation or the decision trail process (Table 3). For the single cross sectional survey, the quality was rated as good by the NIH scale.

**Table 3:** Study Quality.

Strategy Identified	Number of Studies	Reference
Study Purpose	7	19, 21, 25, 28–30, 33
Ethnography	7	19, 25, 28–30, 32, 33
Phenomenology	7	19, 21,25, 28–30, 33
Grounded Theory	1	20
Participatory Action Research	3	25, 28, 30
Types of Reasoning Involved	2	21,25
Theoretical Perspective Included	0	
Sampling done to Saturation	1	25
Constant Comparison	1	25
Inductive and Collaborating findings	6	19, 25, 28–30,33
Decision Trail developed and rules of analysis reported	3	19, 30, 33
Adequate data transformation process into themes/codes	7	19, 21,25, 28–30, 33
Triangulation Reported	6	19, 21, 24, 28–30

### Barriers and Facilitators to Diabetes Management

The first and second order constructs for the barriers and facilitators for health care system interactions, physical activity, diet, and medication adherence are presented below and summarized in Table 4.

**Table 4:** Patient Perceptions of Barriers and Facilitators in Diabetes Management.

Diabetes Management	Barrier	Facilitator	Reference
Health Care Worker Interaction	Patient autonomy	Bicultural, bilingual interpreters	18,19, 21, 25–27, 36, 37
	Lack of time with HCW	Family support	
	Lack of empathy, reassurance by HCW	HCW trusted authority figures	
	Difficulty disclosing issues around management and non-compliance		
Engaging in Physical Activity	Misconceptions of physical activity (harmful)	Needed to be enjoyable, social and culturally specific	21, 22, 26, 27, 29, 31–34
	Lack of motivation, fatalism	Gender specific facilities	
	Culturally inappropriate facilities		
Adopting a Diabetic Diet	Little specific information on dietary changes	Family Support	20,21,23–25,27,29–31,33,34,37
	Misconceptions on what is a healthy diet	Specific information on traditional diet (portion, cooking method)	
	Cultural events and socializing was a deterrent to dietary adherence		
Diabetes Medication Taking	Lack of understanding of role of medications and medication taking behaviors		18–20,23,27,28,33
	Concern for long term safety of medications		
	Preference for phytotherapy and folk remedies		

HCW refers to health care worker.

#### Patient interactions with the healthcare system

Eight studies reported patient views on interactions with health care workers. Most participants trusted their healthcare provider [18,25–27,36]. Despite this trust, participants also reported that language and communication discordance was a significant barrier in receiving information and understanding information on diabetes management [18,19,25–27,29]. Translation services were only intermittently provided and there was a reluctance to use interpreters from the participants’ own community [18]. Patients did not want to burden family members to act as translators [18,24], although some identified they preferred family members to act as translators as it would serve to reinforce the doctor’s advice when the patient was at home. South Asian patients considered the physician as the authoritative source of diabetes knowledge and management [18,25–27, 36] as “physicians were the best people to control glucose” [21]. There were mixed reporting of willingness and confidence to partake in self-management [25,26]. Some South Asian participants did not prefer self-management or autonomy and viewed self-efficacy negatively as they appreciated an authoritative approach [25,26]. Physician’s advice was viewed as superseding advice from others (e.g. family members, dieticians, nurses). In one study [36], 77% of patients reported their preferred source of advice was their family physician, whereas 4% preferred advice from dieticians and 1% from families or friends. Patients also found it challenging to disclose to physicians if they failed to comply with physician recommendations [25]. Although South Asian participants generally appreciated and relied on physicians to provide diabetes advice, they noted a lack of time with physicians and lack of empathy and reassurance from the physicians as a significant barrier to diabetes management [18,25]. Health care workers were seen as sometimes not being able to understand the patient’s economic, social and cultural factors that influenced the patient’s ability to manage their diabetes [21]. Patients also reported transportation difficulties, childcare and lack of available persons to accompany them to appointments as barriers to seeking physician care [2]. Patients in Western countries found that allied healthcare workers were better able to understand their needs and concerns since they had more time available instead of the limited time with the physician [18]. Patients in India found it easier to access their family physician versus dieticians, and were highly likely to follow a suggested diet plan [36].

#### Engaging in physical activity

There were 9 studies that included perceived barriers and facilitators to increased physical activity. The most common barriers included lack of motivation, lack of knowledge, and misconceptions on physical activity. Some South Asian participants held fatalistic beliefs around their ability to prevent diabetes complications [21,26,29,37]. In one study, exercise was thought to have little cultural meaning especially in the context of health and fitness [29]. Moreover, exercise was considered as potentially exacerbating illness or weakness [29, 34] and especially the elderly, should ‘just rest’. Some patients perceived that shortness of breath with increased levels of exercise was a sign of illness, therefore the patients often stopped engaging in physical activity [29]. There was anxiety surrounding safety and security for women when exercising outside of the home in addition to a lack of confidence to attend exercise classes [22,25,29–32]. There was also low understanding of health awareness and particularly, how to incorporate physical activity into their regular lifestyle. Some participants reported a difference in body image perception where a larger body size was considered more prosperous and healthy in this population. Other barriers to physical activity include cold weather, cost of exercise programs, fear of injury with exercise, and transportation difficulties [27,32]. Another significant barrier to physical activity was competing demands and lack of culturally appropriate exercise options [32]. Participants reported that motivators for exercise were to make exercise more enjoyable and role modeling from their own community. A social and cultural focus was also needed to improve their levels of exercise. Participants suggested bhangra dance, sex specific exercise classes and walking groups with friends as enjoyable and culturally appropriate physical activities [31–33].

#### Adopting a diabetic diet

Twelve studies included patient views on adopting a diabetic diet. Modifying the South Asian diet was most often considered difficult in diabetes management by South Asians given the central role of diet in South Asian culture [24]. Food played a key role in maintaining relationships with others and there was considerable social pressure to eat especially during social events [23,24]. Advice to avoid some traditional food items and devaluing the South Asian diet by health care workers as harmful was thought to act as an impediment to adopting dietary advice [27]. The most common barriers to adopting a healthy diabetic diet were lack of awareness on the components and quantities of that diet, general misconceptions on diet, and competing cultural expectations. The South Asian traditional diet typically has high levels of saturated fats, mainly from ghee, milk and yoghurt. Many patients reported not having enough specific details on what types of food to consider that would still be culturally appropriate [23,25,27,34,37]. Participants did not know how to respond to high glucose levels in their diet [37]. Participants believed they should avoid ‘strong foods’, including white sugar, lamb, beef, ghee, solid fat, and spices; and increase consumption of ‘weak foods’ such as boiled rice and cereals to improve their glycemic control [29]. South Asians, especially women, relied on traditional foods and used their traditional diet as a means of treatment for diabetes [20,21,33]. Examples of phytotherapy included using ‘karela’ (bitter gourd), okra, and grapefruits for glycemic control [21]. However, participants reported that healthcare providers were uninformed on whether or not the individuals were eating traditional foods with the purpose of improving hyperglycemia in addition to the prescribed medication [21]. Men and women did appear to have similar knowledge of food values [21, 29]. Both men and women found it challenging to adhere to their diabetic diet during social visits, weddings and extended travel to South Asian countries [21,24,33]. Some participants stated that families were an important source of support as well for maintaining the diabetic diet at home [31,33] whereas others felt family members were occasionally too strict in enforcing a diabetic diet or that not having family or social obligations led to having to eat less [30].

#### Medication taking behavior

Eight studies examined patients’ views on the barriers medication taking or medication adherence and none of the studies included facilitators. There was a lack of understanding that diabetes was a chronic condition that required ongoing and escalating medication therapy [18]. In a study of Bangladeshi patients, there was little impetus to take medicines to reduce diabetes-related mortality given South Asian’s tendency to have fatalistic views [23]. There was reluctance to initiate diabetes medications as South Asian patients felt this signaled their diabetes had deteriorated and they would be identified as “sick” [28]. One participant said; “If you start taking them, you become a patient.” [28] Although participants acknowledged the importance of taking diabetes medications, some participants stated that they deliberately and routinely reduced the amounts of medications. They believed these medications worked instantly and when patients felt they did not need them, or when they ate less, they did not need to be taken. Some participants reduced or missed dosages to avoid short-term side effects or when travelling overseas [27,28]. There was also a general concern that taking diabetes medications long term would be harmful [28]. There was considerable preference and usage of ayurvedic and phytotherapy as these treatments were believed to be effective but considerably safer than western medication [19, 20, 33]. South Asians were also reluctant to disclose to their healthcare worker if they were missing or reducing their diabetes medications [28].

#### Differences in barriers and facilitators in South Asians living in western countries and India

In comparing findings in studies conducted in Western countries and India, most barriers were consistent across countries. Preference for traditional therapies, perceptions that exercise depletes energy, and a priority to keep families well fed were similar among South Asians living in the West and India. Both groups considered the physician as the main source of information, physicians as an authority and described poor self-efficacy in diabetes self-management. However, patients living in India reported that they only sought care when they felt they had a problem and avoided routine diabetes care because of high costs. South Asians living in the West viewed lay sources of knowledge as a major influence on their behavior, difficulties with transportation, access to health care, and perceived diabetes care (physician visits etc.) as being a burden on their family. Barriers reported only among South Asians living in the West additionally included cold climate, language barriers and that advice was not considered culturally appropriate or specific enough to operationalize. Some South Asians living in the West also felt that their diabetes was better controlled when they would return to their native country.

### Overarching Themes

Two overarching themes emerged from review of patient’s reported barriers from the primary studies: 1) lack of knowledge and prevailing misconceptions, and 2) lack of culturally specific management to effective diabetes management.

#### Lack of knowledge and misconceptions

Lack of knowledge was a theme pervasive across all aspects of diabetes management. Lack of specific and concrete culturally appropriate information was considered a significant barrier to diabetes knowledge. Language barriers and also preferences for information and direction from physicians were limiting factors for receiving knowledge. Receiving knowledge through families as translators had some noted limitations. Media, specifically television and newspapers were considered helpful sources of knowledge for women working inside the home [19].

Along with lack of knowledge, there were misconceptions within diabetes management as well as each component of diabetes management. Preventative care was not well understood as patients had a passive or fatalistic view towards their prognosis. Several common misconceptions included that exercise led to deteriorating health, understanding on what foods were appropriate for a diabetic diet, and medication taking behaviors.

#### Lack of culturally specific chronic care modifications

Throughout the components of diabetes care, there was a reported need for understanding of the South Asian culture and its impact on diabetes management. Dietary advice needed to be centered on concrete examples of South Asian traditional foods and ingredients. The role of western medications in addition to folk remedies also needed to be discussed. Sex specific facilities for exercise or safe environments for exercise were viewed as important especially for women [26]. Many of these barriers were more prevalent among South Asians living in Western countries.

## Discussion

Our literature review revealed multiple experienced or perceived barriers and few facilitators to diabetes management in South Asian populations. The barriers and facilitators were generally similar among South Asian patients with diabetes living in Western countries and in South Asian countries, however with a few differences. The key themes across many aspects of diabetes management included lack of knowledge, prevailing misconceptions, and lack of culturally specific management as barriers to effective diabetes management. There were few studies that identified facilitators to management, but culturally appropriate strategies and family support was identified across various components of diabetes management.

Similar to South Asian participants in this review, limited knowledge of diabetes and language discordance between patient and provider are barriers to diabetes self-management in the general population, and in other non-English speaking ethnic groups [38]. Misperceptions of diabetes management are also common among ethnic groups. Some Hispanic Americans reported misconceptions that insulin therapy was harmful [39], held fatalistic beliefs, used folk healers and alternative treatments for diabetes management [40]. Culture also played a role in willingness to adhere to diabetes management. Family needs are considered paramount and adhering to a treatment regimen that takes time or resources away from family responsibilities is considered self-indulgent to Latino patients. In Chinese culture, the freedom to enjoy food is an essential component in one's quality of life [41].

The barriers identified for diabetes were also similar for other chronic diseases among South Asians. In a qualitative study of 91 participants of the Khush Dil program originating from Pakistan, Bangladesh and India living in Edinburgh, participants reported competing priorities, social pressures to not change their diet and stress as barriers to adopting a heart healthy diet and physical activity [42]. Barriers reported among South Asians to attend cardiac rehabilitation programs or cardiac prevention programs included lack of transportation, a sense of fatalism, the harmful effects of exercise, and language barriers [43]. Facilitators included family support, and spoken content rather than written literature [43]. There are few studies evaluating the effectiveness of addressing these barriers and facilitators for chronic disease management in South Asians. In a qualitative precede-proceed study in Canada to improve cardiac rehabilitation attendance by addressing barriers in South Asians, investigators stressed the importance of physician endorsement of the program given the strong emphasis that South Asians place on the physician as the major authoritative source of knowledge and expertise, making programs more accessible (provided transportation, accessible hours), family and community support that leverages the collectivism within the South Asian culture, caring staff and ongoing support from physicians. These factors were considered by the South Asian patients to account for their improved adherence and participation in cardiac rehabilitation programs [44].

### Challenges In Developing Culturally Tailored Diabetes Management

Culturally tailored diabetes education programs have been shown to improve diabetes targets such as A1c compared to programs that were not culturally tailored in Hispanic and African-American patients [45]. Although diabetes education and other health interventions targeted to South Asian patients rely on bicultural and bilingual delivery of health information, from this review, the lack of knowledge and understanding suggests deeper cultural adaptations, aimed at addressing the groups’ cultural beliefs, knowledge and understanding are also needed [46]. Although diabetes education programs in western countries are heavily grounded in patient centeredness and empowerment, some South Asian patients may not actively follow these ideals, since they rely on tradition and authority [25, 26]. There is still low motivation for patients to become partners with healthcare providers to address diabetes management in part due to beliefs in fatalism. Health programs that include deeper cultural adaptations, addressing the cultural beliefs and culture based understanding of disease including poor understanding of the natural course and chronicity of disease and beliefs in fatalism may begin to help improve disease management. The importance of empathy, care, cultural sensitivity and clarity of advice from health care workers may also improve diabetes management in this group.

Changing diet in the South Asian community is likely difficult given the deep cultural significance of food and the important social role of diet in maintaining social relationships and tradition. Barriers to diet can be addressed through increasing the individual’s knowledge and awareness of what traditional foods are appropriate with additional specific details on portion size and cooking methods. A potential facilitator in dietary change was increasing family support and creating family level changes in the household diet. However, family involvement in diabetes prevention and weight loss studies in South Asian patients have not shown any benefit compared to care without family involvement [47].

A major component in achieving glycemic control in diabetes is through physical activity. Knowledge translation strategies would need to address culturally- based concerns including dispelling fears of injury or safety and overcoming the predominant view that elderly people should ‘just rest’. As regular exercise is not a significant part of South Asian culture, improving participation using peer or community role models, and readily available exercise facilities in local areas with a high South Asian population might mitigate reluctance to incorporate physical exercise into daily routines. These facilities would need to leverage gaining social networks and have a culturally appropriate social element (e.g. dance for some South Asian groups) which was considered a high priority for South Asian patients. Some of the other barriers to physical activity include the structural landmark of the facilities and activity sessions, such as gender specific especially women-only facilities. This type of implementation may help to address the lack of confidence to attend exercise class as especially reported by Muslim women.

Patients of South Asian descent in both western countries and South Asian countries appear to use both biomedicine and aryuvedic medicine in addition to self-therapy [20]. Although the use of both types of medications varies depending on the duration of diabetes, many of the patients hope to delay their dependence of bio medicinal drugs by using natural therapies such as herbs, and homeopathy [20]. Many of these patients find aryuvedic and plant therapy to be safer due to fewer perceived side effects [20]. Improving medication adherence in South Asian patients would also have to address fundamental misconceptions that diabetes medications need to be taken regularly to extract the maximum benefit and that diabetes is a chronic condition that often requires lifelong medications to prevent complications. Addressing concerns of long-term side effects against long-term benefits would also need to be discussed. Working with patients to incorporate both phytotherapy and western medications may be a culturally appropriate strategy to enhance adherence.

Although many barriers and facilitators were consistent among South Asians living in India and the West such as knowledge deficits and misperception, not surprisingly, the lack of cultural adaptation were reported by South Asians living in Western countries. These findings suggest that some interventions that focus on knowledge deficits and misperceptions may be translatable across countries, but appropriate cultural adaptation is still required in the West.

### Limitations

There were several limitations in this study. There was a lack of information on facilitators for medication compliance, and lack of comparisons between ethnic groups. We were unable to stratify the results by sub-groups (e.g. elderly vs. younger) as these groups may have differing beliefs. Finally, although this review included 1980 participant views, the reported barriers and facilitators may not be representative within subgroups of the South Asian population with diabetes.

## Conclusion

Our review, demonstrated several recurring themes of lack of knowledge, prevailing misconceptions and lack of culturally tailored diabetes management as key barriers to diabetes management from South Asian patient’s view. Culturally appropriate programs that focus on improving communication; discussing common misperceptions in the South Asian community on diabetes management and leveraging cultural beliefs and family as a resource may help to improve diabetes control.
